# Effects of *Agaricus bisporus* Mushroom Extract on Honey Bees Infected with *Nosema ceranae*

**DOI:** 10.3390/insects12100915

**Published:** 2021-10-07

**Authors:** Uros Glavinic, Milan Rajkovic, Jovana Vunduk, Branislav Vejnovic, Jevrosima Stevanovic, Ivanka Milenkovic, Zoran Stanimirovic

**Affiliations:** 1 Faculty of Veterinary Medicine, University of Belgrade, Bulevar Oslobodjenja 18, 11000 Belgrade, Serbia; mrajkovic@vet.bg.ac.rs (M.R.); branislavv@vet.bg.ac.rs (B.V.); rocky@vet.bg.ac.rs (J.S.); zoran@vet.bg.ac.rs (Z.S.); 2 Faculty of Agriculture, Institute for Food Technology and Biochemistry, University of Belgrade, Nemanjina 6, 11080 Belgrade, Serbia; vunduk@agrif.bg.ac.rs; 3 Ekofungi Ltd., Padinska Skela bb, 11000 Belgrade, Serbia; ekofungi@gmail.com

**Keywords:** honey bee, *Nosema ceranae*, mushroom extract, *Agaricus bisporus*, immune-related gene expression

## Abstract

**Simple Summary:**

*Nosema ceranae* affects honey bee (*Apis mellifera* L.) causing nosemosis disease that often induces serious problems in apiculture. Antibiotic fumagillin is the only licenced treatment against nosemosis, but its effectiveness is questioned and its usage associated with risk of bee mortality and appearance of residues in bee products. In search for alternative treatment for the control of nosemosis, water crude extract of *Agaricus bisporus* was tested on bees in laboratory (cage) experiments. Bee survival and food consumption were monitored together with *Nosema* infection level and expression of five genes (abaecin, hymenoptaecin, defensin, apidaecin, and vitellogenin) were evaluated in bees sampled on days 7 and 15. Apart from the gene for defensin, the expression of all tested genes was up-regulated in bees supplemented with *A. bisporus* extract. Both anti-*Nosema* and immune protective effects of *A. bisporus* extract were observed when supplementation started at the moment of *N. ceranae* infection or preventively (before or simultaneously with the *Nosema* infection).

**Abstract:**

*Agaricus bisporus* water crude extract was tested on honey bees for the first time. The first part of the cage experiment was set for selecting one concentration of the *A. bisporus* extract. Concentration of 200 µg/g was further tested in the second part of the experiment where bee survival and food consumption were monitored together with *Nosema* infection level and expression of five genes (abaecin, hymenoptaecin, defensin, apidaecin, and vitellogenin) that were evaluated in bees sampled on days 7 and 15. Survival rate of *Nosema*-infected bees was significantly greater in groups fed with *A. bisporus*-enriched syrup compared to those fed with a pure sucrose syrup. Besides, the anti-*Nosema* effect of *A. bisporus* extract was greatest when applied from the third day which coincides with the time of infection with *N. ceranae.* Daily food consumption did not differ between the groups indicating good acceptability and palatability of the extract. *A. bisporus* extract showed a stimulative effect on four out of five monitored genes. Both anti-*Nosema* and nutrigenomic effects of *A. bisporus* extract were observed when supplementation started at the moment of *N. ceranae* infection or preventively (before or simultaneously with the infection).

## 1. Introduction

The honey bee (*Apis mellifera* L. (Hymenoptera: Apidae)) is a pollinator important for agricultural production [1] and providing essential macro- and micronutrients [2]. Considerable colony losses in the United States and Europe have significant negative economic and environmental consequences, and can be caused by many factors, most often pathogens and parasites, environmental pollution, nutritional stress, and inadequate beekeeping management [3,4,5].

One of the most common diseases of honey bees is nosemosis caused by microsporidia of the genus *Nosema* [3] with three species that may affect *A. mellifera: N. ceranae, N. apis*, and *N. neummani.* Currently, *N. ceranae* is one of the most widespread microsporidium causing infections in honey bees [6] with the different symptoms compared with those induced with *N. apis* [7]. *N. ceranae* is primarily a pathogen of honey bee midgut. However, this endoparasite or its DNA was also found in other tissues [8,9,10] and hemolymph [11]. Pathological changes in the epithelial cells of the midgut lead to digestive and metabolic disorders causing malnutrition in adult bees [12], diarrhea, decreased honey production, and increased mortality in winter bees [13]. There are reports that *N. ceranae* induces changes in the carbohydrate metabolism [14,15,16,17], which may increase nutritional and energetic stress [3,15,16,17,18,19,20]. Infection with *N. ceranae* compromises immunity [21] and induces oxidative stress in bees [5], especially in combination with pesticides [22,23]. It has also been shown that infection of bees with *Nosema* spp. can reduce the effectiveness of acaricides used for the control of honey bee mite *Varroa destructor* [24]. In addition to bee diseases, many problems in beekeeping are caused by chemical substances used in the control of bee pathogens due to the many side effects on adult worker bees (increased mortality, shortened lifespan, down-regulation of immune-related genes, induction of toxic stress and up-regulation of detoxification-related genes, decreased worker mobility, learning, memory and trophallaxis, increased *Nosema ceranae* infection rate), bee brood (increased egg, larval and pupal mortality, reduced brood survival and brood area, delayed larval development, delayed adult emergence), reproduction (reduced drone production, longevity and weight, decreased number and viability of drone sperm, increased queen mortality, failure in queen’s development, reduced queen mating success, queen pupal weight, queen adult weight, queen cell acceptance), consequently leading to weakening of bee colonies and increase in susceptibility to diseases even if used at recommended doses (reviewed in [25]).

The control of nosemosis includes a wide range of beekeeping practices [3] and application of chemicals, such as fumagillin, an antibiotic derived from the fungus *Aspergillus fumigatus* [26,27]. Although fumagillin use is prohibited in most of European countries due to risk of its commercial formulations (Fumagilin-B, Fumidil-B,) to leave residues in bee products [28,29,30], to affect food safety [31,32], and to increase bee mortality [30], however, it is still licensed in the United States [33], Canada [34], and Argentina [35]. Moreover, its effectiveness has been questioned [28], imposing a constant effort of scientists to discover alternative substances, which would be effective against *Nosema* spp. Among many natural compounds tested [5,36,37,38,39,40,41,42,43,44,45,46,47], some of them showed promising anti-*Nosema* effects such as algae and fungus extracts [5,22,36,37,38,39], chitosan and peptidoglycan [40], naringenin, sulforaphane and carvacrol [41], probiotics [42,46], natural substances from Brassicaceae defatted seed meals [43], and natural-based commercial formulations [5,44,47,48]. Diet with the addition of plant-based, amino acid and vitamin supplements, led to reduction of *N. ceranae* spore number [5,48], immunostimulation, and reduction of oxidative stress in *N. ceranae*-infected bees [5], and consequently to the improved condition of bees. Interest in mushrooms has been increasing among researchers due to their nutritional and medicinal properties; mushrooms of the genus *Agaricus* contain numerous biologically-active substances (such as glucans, mannan, lentinan, schizophyllan, scleroglucan) with certain antitumor, antidiabetic, and immunostimulatory effects [49,50,51,52,53,54,55,56]. Stevanovic et al. [38] observed a favorable effect of *A. blazei* extract on the strength parameters of bee colonies in a field experiment, while Glavinic et al. [5] revealed the positive effect of *A. blazei* extract in laboratory experiment. The extract stimulated the expression of genes important for immunity, reducing oxidative stress caused by *N. ceranae* and consequently reducing *N. ceranae* infection. Beneficial effects on honey bees were observed for other mushroom species, too; extracts derived from the mycelia of *Fomes fomentarius*, *Ganoderma applanatum*, *G. Resinaceum*, and *Trametes versicolor* demonstrated strong anti-viral efficacy against two bee viruses (deformed wing virus—DWV) and Lake Sinai virus—LSV) in both laboratory and field trials [57].

Having in mind the described positive effects of *A. blazei* on honey bees and its potential in *Nosema* control, we decided to assess the effect of *A. bisporus* extract on bee survival, *Nosema* infection, and the expression of genes important for bee immunity in a laboratory experiment. *A. bisporus* is the most cultivated mushroom species among Western countries which makes this research and its application in beekeeping more cost-effective compared to the research and application of *A. blazei*.

## 2. Materials and Methods

### 2.1. Agaricus bisporus Extract Preparation

Water extract of the commercially-cultivated white button mushroom (*Agaricus bisporus*, strain A15) was prepared according to the procedure described by Klaus et al. [58]. In summary, dry powdered fruit bodies of mushrooms were extracted with distilled water at 121 °C, 1.2 bar, for 60 min. After being filtered, the liquid part was evaporated up to 1/3 of its volume and precipitated with 96% ethanol overnight. The material was then centrifuged, dried at 40 °C, powdered, and kept in the refrigerator (RK6333W, Gorenje, Velenje, Slovenia) before analysis.

### 2.2. Bees and the Design of Cage Experiment

During April 2020, frames containing areas with sealed brood were taken from three honey bee colonies at the experimental apiary of the Faculty of Veterinary Medicine, University of Belgrade. The frames were placed in net bags (to prevent the dissipation of hatched bees) and kept overnight in a preset incubator (Inkubatori, Pozarevac, Serbia) at the temperature 34 ± 1 °C and humidity 66 ± 1%. The next morning, the newly emerged worker bees were collected from the frames and transferred to experimental cages (60 bees per experimental cage: 10 bees were sampled for the RNA extraction, 20 for *Nosema* spore counting and the remaining 30 bees served for monitoring the survival) and each group comprised three cages (replicates). Cages were specifically designed by Glavinic et al. [21] for this experiment. Briefly, a plastic jar was punched to allow entry of air, placed with the lid down, and equipped with a plastic strainer inserted in the center of the lid allowing bees to take the syrup from the small petri dish placed below. The whole experiment was repeated, and the results were merged into a single dataset.

### 2.3. The Selection of an Appropriate Extract Concentration

To determine the optimal concentration of the extract, only the survival of bees was monitored in the first part of the experiment. Newly emerged bees were divided into four experimental groups. The control group (C group) received pure sucrose syrup (50% *w*/*v*). The remaining three groups (Table 1) received sucrose syrup (50% *w*/*v*) supplemented with *A. bisporus* extract at concentrations of 100 µg/g (Abs-100 group), 200 µg/g (Abs-200 group) and 400 µg/g (Abs-400 group). The experiment lasted 15 days. The dead bees were counted and removed from the cages on a daily basis.

### 2.4. Effects of A. bisporus Extract on Nosema Infection

In the second part of the cage experiment, the effect of *A. bisporus* extract at a concentration of 200 µg/g was evaluated. Six groups were established and all were fed with 50% (*w*/*v*) sucrose syrup. There were two control groups: non-infected (NI) and infected with *N. ceranae* (I). The remaining four experimental groups received sucrose syrup enriched with *A. bisporus* extract either from the first day (in group Abs, those are non-infected and in *Nosema*-infected, group I-Abs1), or from the third or sixth day (groups I-Abs3 and I-Abs6, respectively, both *Nosema*-infected (Table 1)).

### 2.5. Inoculum Preparation, Experimental Infection, and Bee Sampling

Inoculum preparation and experimental infection of bees were performed according to the previously described methodology [5,21]. Briefly, the inoculum was prepared by crushing the abdomens of *N. ceranae*-infected bees in distilled water. Number of spores were determined according to Cantwell [59]. Freshly-prepared spore suspension with 99% viability, assessed with 4% trypan blue (Sigma–Aldrich, Steinheim, Germany), mixed with 50% sucrose (Centrohem, Stara Pazova, Serbia) solution was used to obtain 1 × 10^6^ spores/mL final concentration. Bees in the infected control group (I) and treated groups (I-Abs1, I-Abs3, and I-Abs6) were infected with *N. ceranae* spores on day 3 [5]. From each cage, on days 7 and 15, bees were sampled for counting *Nosema* spores and gene expression analysis (Table 1). The remaining bees from the cage were used to determine the survival rate. Dead bees were removed from the cage and counted daily. During the experiment, syrup consumption was measured by weighing the food before and after the bees had been fed for 24 h [60]. According to these data, average consumption/bee/day was calculated.

### 2.6. Nosema Spore Counting

On the day of sampling (day 7 and day 15), 10 bees were taken from each cage. The abdomen of each bee was placed in a 1.5 mL tube and macerated in 1 mL of distilled water in Tissue Lyser II (QIAGEN, Hilden, Germany) for 1 min at 25 Hz. The number of *N. ceranae* spores was determined using a hemocytometer described in Cantwell [59] and OIE [61], and used also in our previous studies [5,21,22,23].

### 2.7. RNA Extraction and cDNA Synthesis

The total RNA was extracted with Quick-RNA MiniPrep Kit (Zymo Research, Irvine, CA, USA). A single bee was placed in a 1.5 mL tube with 500 μL of Genomic Lysis Buffer. Homogenization was performed in a TissueLyser II (QIAGEN, Germany) for 1 min at 25 Hz using a 3 mm tungsten carbide bead (Qiagen, Hilden, Germany). Other extraction steps were performed according to the manufacturer’s instructions. In-column DNase treatment (treatment with DNase I Reaction Mix) was applied for all samples during the extraction process with the aim to remove contaminating DNA. cDNA was immediately generated from extracted RNA using the RevertAid™ First Strand cDNA Synthesis Kit (Thermo Fisher Scientific, Vilnius, Lithuania).

### 2.8. Real-Time Quantitative PCR

SYBR green method was used for real-time PCR (qPCR) amplification in a 20 µL reaction mixture with “FastGene^®^ IC Green 2 x qPCR Universal Mix” (Nippon Genetics, Düren, Germany) according to instructions of the manufacturer and modifications described by Glavinic et al. [5]. A specific primer pair was used for each gene (Table 2). The qPCR reactions were performed in a Rotor-Gene Q 5 plex (Qiagen, Valencia, CA, USA). Gene expression level of abaecin, hymenoptaecin, defensin, apidaecin, and vitellogenin was determined using the 2^−ΔΔCT^ method described in Galvinic et al. [5] while β-actin was used as an internal control for normalization of each gene expression.

## 3. Statistical Methods

The survival of bees was monitored by the number of dead bees per day in each experimental group. The data on the survival distribution obtained in the Kaplan–Meier survival estimator were compared in the log-rank test. The results for *N. ceranae* spores and gene expression were tested for normality by using Shapiro–Wilk’s test. Due to the normal distribution of data (Shapiro–Wilk’s test, *p* > 0.05), groups were compared in two-way ANOVA with repeated measures in one factor, followed by Tukey’s test within and Sidak’s test between groups over time. The levels of significance below 0.05 (*p* < 0.05) were considered significant. The analyses were done using GraphPad Prism 7.0 (GraphPad Software, San Diego, CA, USA).

## 4. Results

### 4.1. Bee Survival and Food Consumption

**Figure 1:**
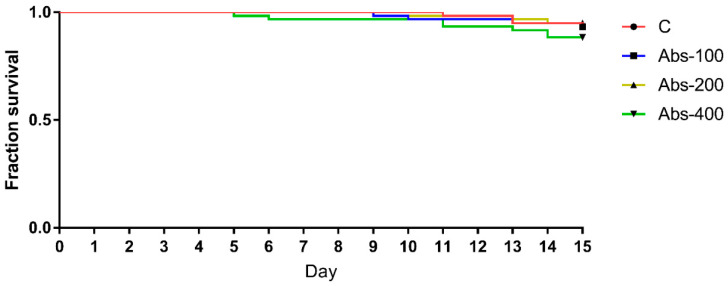
Survival of bees treated with *A. bisporus* extract: 100 µg/g (Abs-100), 200 µg/g (Abs-200), 400 µg/g (Abs-400), and control (C) group.

In the first part of the experiment, which was conducted to determine the optimal concentration of the extract for further investigation, no significant differences in bee survival (*p* > 0.05) were detected between all groups (Figure 1). The lowest number of dead bees was in the group treated with 200 µg/g of *A. bisporus* extract (Abs-200). Consequently, this concentration was used in the second part of the experiment.

**Figure 2:**
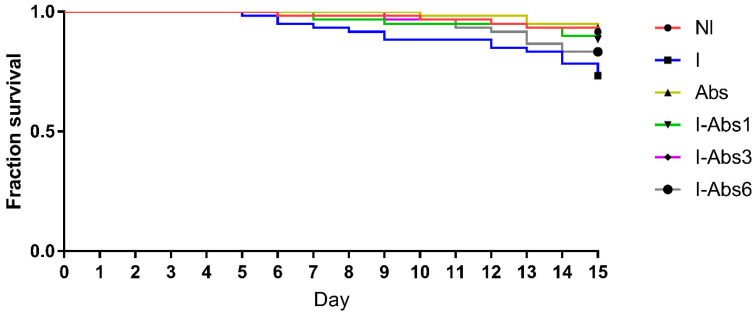
Survival of bees infected with *N. ceranae* (I), non-infected but treated with *A. bisporus* extract (Abs), bees infected with *N. ceranae* and treated with *A. bisporus* extract from day 1 (group I-Abs1), day 3 (I-Abs3), and day 6 (I-Abs6), as well as bees from the control non-infected (NI) group.

The number of dead bees in the second part of the experiment (Figure 2) was significantly higher in the infected control group (I) compared to NI (*p* = 0.009), Abs (*p* = 0.003), I-Abs1 (*p* = 0.036), I-Abs3 (*p* = 0.018), and not significantly different (*p* = 0.185) in comparison with group I-Abs6 (log-rank test). The survival of bees in the non-infected control group (NI) did not differ statistically compared to the groups of Abs (*p* = 0.714), I-Abs1 (*p* = 0.552), I-Abs3 (*p* = 0.760), and I-Abs6 (*p* = 0.173).

There were no differences in daily food consumption between the control group and all treatment groups. Mean consumption across all groups was 121.63 mg/bee/day (Figure S1).

### 4.2. Quantification of N. ceranae Spores

**Figure 3:**
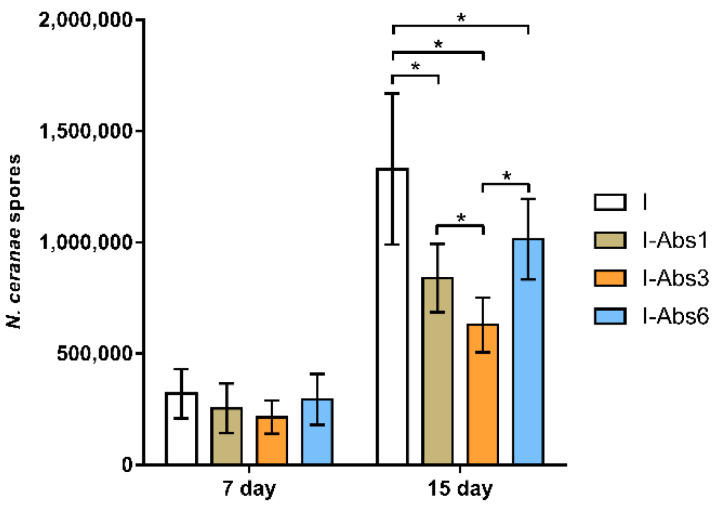
Number of *N. ceranae* spores in infected group (I) and groups of bees infected with *N. ceranae* and treated with *A. bisporus* extract from day 1 (I-Abs1), day 3 (I-Abs3), and day 6 (I-Abs6). * *p* < 0.05.

In samples of bees from the control non-infected group (NI) and non-infected but treated with *A. bisporus* extract (Abs), no spores of *N. ceranae* were detected at any time of sampling. By comparing the number of spores in groups infected with *N. ceranae* on the 7th and 15th day of sampling, Shidak’s multiple comparison test showed a significantly higher (*p* < 0.001) number of spores on day 15 compared to day 7. In bee samples from day 7 of the experiment, the Tukey test revealed no significant differences in the number of spores among all groups (*p* > 0.05). In contrast, on day 15 (Figure 3), the number of spores was significantly higher in group I compared to all other groups (*p* < 0.001). The lowest number of spores was detected in group I-Abs3, significantly lower than in groups I and I-Abs6 (*p* < 0.001) and I-Abs1 (*p* < 0.05).

### 4.3. Gene Expression Analyses

**Figure 4:**
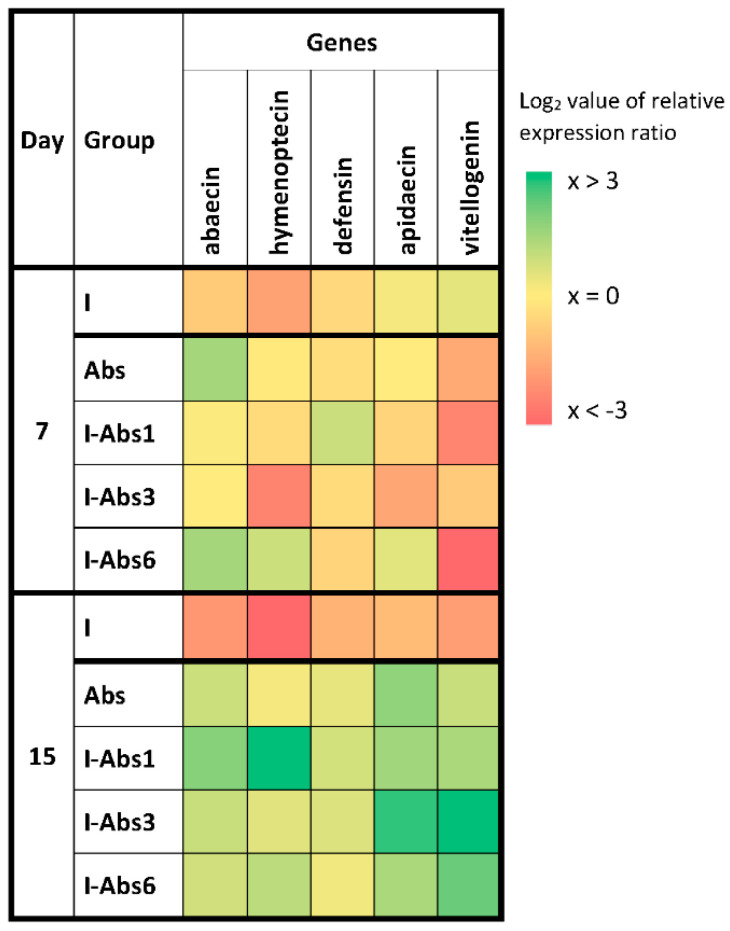
Heatmap of immune−related genes (means of Log2 of relative expression ratios for abaecin, hymenoptaecin, defensin, apidaecin and vitellogenin) at different time points in experimental groups. *N. ceranae* infected control (I), group treated with extract (Abs), and groups infected and treated with *A. bisporus* extract from day 1 (I-Abs1), day 3 (I-Abs3), and day 6 (I-Abs6). Range log2 value of relative expression ratio is given in the legend on the right indicating up- or down-regulation of the expression. Group names are indicated in Table 1.

**Figure 5:**
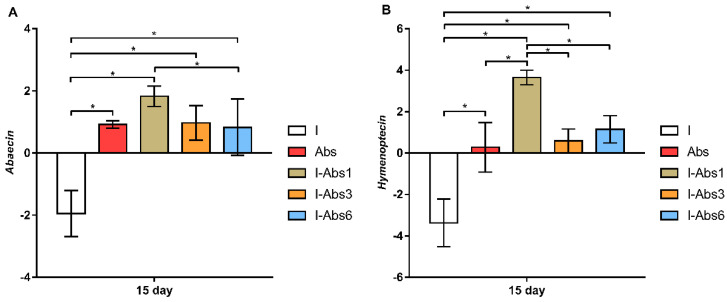

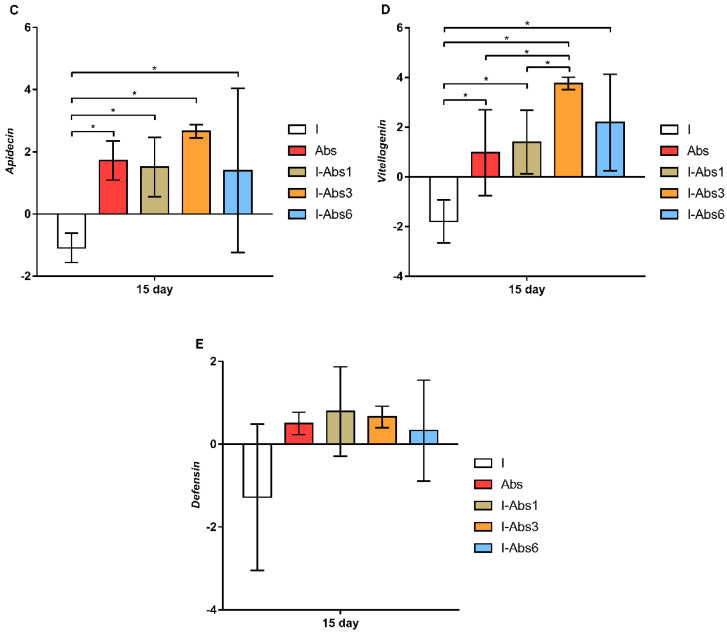
Gene expression levels for abaecin (**A**), hymenoptaecin (**B**), apidaecin (**C**), vitellogenin (**D**), and defensin (**E**) in bees infected with *N. cerane* (I), non−infected but treated with *A. bisporus* extract (Abs), as well as bees infected with *N. cerane* and treated with *A. bisporus* extract from day 1 (I-Abs1), day 3 (I-Abs3) and day 6 (I-Abs6) of the experiment. * *p* < 0.05.

On day 7, the highest expression levels for the majority of monitored genes were in groups Abs and I-Abs6, in contrast to the infected control (I) group where the lowest gene expression was detected (Figure 4). However, the most important and most significant changes in expression levels were obtained on day 15 (Figure 4 and Figure 5). According to the Tukey test, abaecin expression levels (Figure 5A) were significantly lower in group I compared to all other groups (*p* < 0.001), but also in group I-Abs6 compared to I-Abs1 (*p* < 0.05). Gene expression for hymenoptaecin (Figure 5B), on day 15 of the experiment, was significantly lower in group I (*p* < 0.001) compared to all other groups. However, pairwise comparisons of all treated groups revealed that the expression of the hymenoptaecin gene was significantly higher in the I-Abs1 group compared to I-Abs3 (*p* < 0.001) and I-Abs6 (*p* < 0.01). The expression of apidaecin (Figure 5C) and vitellogenin (Figure 5D) was also found to be lower in the control infected group (I) compared to all other groups (*p* < 0.01). The expression of the gene for apidaecin did not differ among the groups treated with *A. bisporus* extract, while the level for vitellogenin was higher (*p* < 0.01) in the group I-Abs3 compared to the groups I-Abs1 and Abs. Regarding gene expression for defensin, although the lowest level was in the group I (Figure 5E), the Tukey test showed no significant differences between the experimental groups.

## 5. Discussion

Nosemosis is a common and widespread disease that negatively affects adult bees [37]. Infection with *N. ceranae* is especially dangerous due to its implied role in colony losses [3]. There is a relatively small number of safe preparations used as alternatives to fumagillin in nosemosis control. Thus, there is a constant effort of scientists to discover new natural substances with beneficial effects in the control of this disease [5,36,37,38,39,40,41,42,43,44,45,46,47].

This is the first study in which *A. bisporus* extract was tested on honey bees. In the first part of the experiment, none of the tested concentrations of *A. bisporus* extract (100, 200, and 400 µg/g) increased bee mortality compared to control. This has been expected, bearing in mind already reported beneficial effects of other *Agaricus* spp. extracts on bees [5,38] as well as of other natural-based dietary supplements [21,48,64,65,66,67]. Despite the absence of significant differences among groups, the lowest number of dead bees was in the group fed with 200 µg/g of *A. bisporus* extract. This result, along with the economic aspect (optimal ratio between the extract amount and the benefit), led us to choose this concentration for further testing. In this experiment, a significantly lower survival rate in the group of infected bees (I) compared to the non-infected group (Figure 2) suggests undoubted fatal effects of *N. ceranae* infection, which is consistent with previous studies [5,21,68,69]. However, feeding bees with the addition of *A. bisporus* extract starting from day 1 and 3 at a concentration of 200 µg/g significantly increased the survival rate of infected bees compared to infected bees (group I) fed without the addition of mushroom extract (Figure 2). This result is in accordance with our previous findings where an extract of related fungus *A. blazei* induced better bee survival [5]. In a field experiment, Stevanović et al. [38] revealed the beneficial impact of *A. blazei* extract on the strength of honey bee colonies. However, when the extract was applied to infected bees starting from day 6, it did not contribute significantly to mortality decrease compared to group I (Figure 2). This finding indicates a better protective-preventive effect (application before or at the moment of infection) compared to the effect obtained by post-infection application. Having in mind that the main action of medicinal mushroom metabolites is immunomodulation [70], this result was expected. In addition, the extract used in this study was rich in polysaccharides (more than 68%) which are the most reported immunomodulatory compounds from mushrooms [71,72]. The absence of significant differences in bee mortality between the control non-infected group (NI) and all groups that received *A. bisporus* extract in the diet (Abs, I-Abs1, I-Abs3, and I-Abs6) is an additional confirmation of the extract’s positive effects on bee survival regardless of the application period, similarly as previously reported for *A. blazei* extract [5], plant extracts (*Aristotelia chilensis*, *Ugni molinae*, *Gevuina avellan*), and propolis [73].

The level of nosema infection was monitored by counting spores on days 7 and 15 of the experiment. The presence of spores in infected groups, and its absence in non-infected groups (NI and Abs) suggest that there was no cross-contamination between the experimental groups, and the design of the cage [21] is adequate for such research. This confirms that the experiment has been performed accurately and in line with COLOSS BEEBOOK recommendations [74]. A significantly higher number of spores was detected on day 15 compared to day 7 in all infected groups. Analysis of the number of spores in the samples collected on day 7 did not reveal significant difference among infected groups. This finding was expected since the infection was still in the initial stage [75]. In contrast, on day 15, a significantly higher number of spores was in group I (Figure 3) compared to all other groups that received the extract. Comparing groups infected with the *Nosema* and treated with *A. bisporus* extract (I-Abs1, I-Abs3, and I-Abs6), the lowest spore number was detected in the group I-Abs3 (Figure 3). Thus, we can conclude that the extract of white button mushroom has a noticeable anti-*Nosema* effect. Moreover, this effect is the highest when applied to start from the third day which coincides with the time of infection with *N. ceranae*. Such effects have already been demonstrated for *A. blazei* [5], algae extract also rich in polysaccharides [37], and other supplements [21,73]. Hayman et al. [76] explained microsporidia reduction by inhibition of adhesion to the target cells. Vunduk et al. [77] also showed that mushroom extract exhibits antiadhesion and biofilm formation effect against foodborne bacteria. The extract’s more beneficial effect in groups IAbs-1 and I-Abs3 could be explained by the absence of infected epithelial cells with *N. ceranae* [75], which gives a better opportunity for more efficient action (adhesion inhibition) of the extract.

Infection with *N. ceranae* suppressed most of the immune-related genes examined in this study (Figure 4 and Figure 5), which is in line with previous reports of the immunosuppressive effect of this endoparasite [5,21,78,79,80]. The expression level of all genes, except defensin, was significantly lower in group I compared to all other experimental groups (Figure 4 and Figure 5). This result is not surprising considering the amount of *N. ceranae* spores detected at the end of the experiment (highest spore load in group I—Figure 3) but also the bee mortality (highest number of dead bees in group I—Figure 2). The gene expression levels in all the groups supplemented with *A. bisporus* extract (Abs, I-Abs1, I-Abs3, and I-Abs6) indicate its stimulatory effect on the expression of all monitored immune genes except defensin (Figure 4 and Figure 5). This finding is consistent with the previous study demonstrating the stimulating effect of *A. blazei* on honey bee immunity [5,39]. Higher levels of immune-related gene expression in infected and treated groups compared to the infected control group (I) indicate the immunoprotective and immunomodulating effect of *A. bisporus* extract. Moreover, higher gene expression levels (Figure 4 and Figure 5) in infected and treated groups, and lower spore loads in those groups (Figure 3) revealed an inverse correlation between spore loads and levels of immune-related gene expression. Gene expression levels on day 15 compared to day 7 were significantly higher for all tested genes in group I-Abs3 (except the one for defensin); significantly higher for all tested genes in group I-Abs1 (except those for defensin and apidaecin); significantly higher only for vitellogenin gene in I-Abs6 group (Figure 4), and significantly lower for all tested genes in the infected control group (I). Greater stimulation of gene expression in group I-Abs1 and I-Abs3 compared to I-Abs6 indicates better efficacy of the extract before and at the moment of infection with *N. ceranae*. A better protective effect is expected as the extract is rich in polysaccharides [5,36,37,38], while the anti-*Nosema* effect is more typical for antibiotics such as fumagillin [5,29,30,33,34,35]. Moreover, we used crude water extract which, besides polysaccharides (almost 70%), contains proteins (5.31%) and phenolic compounds (2.7%) as well [71,81]. These metabolites express different modes of action. Proteins are often bound to polysaccharides and cumulative immunomodulatory effect can be expected. Phenolics, as fungal secondary metabolites, mainly act as antioxidants, and can be important in the early stage of infection development [82].

## 6. Conclusions

This is the first study of the effects of *A. bisporus* extract on honey bees that showed the potential for improving the survival and the immunity of bees infected with *N. ceranae*. The effect on immunity has been demonstrated through increased expression of immune-related genes in both non-infected bees and bees infected with *N. ceranae*. Besides, *A. bisporus* extract was effective in reducing the number of *Nosema* spores. The best results were observed when the extract was applied at the moment of infection or preventively (before or simultaneously with the *Nosema* infection). Finally, the tested extract has a strong stimulatory effect on gene expression in both *N. ceranae*-infected and uninfected bees. In the absence of adequate and safe natural-based therapy for nosemosis, we can conclude that *A. bisporus* extract has a great potential for use in the control of this bee disease and needs to be further investigated in the field experiments.

## Figures and Tables

**Table 1:** Experimental design.

GROUP ^1^	Day of Starting the Treatment ^2^	*N. ceranae* Infection Day ^2^	Sampling Day ^2^	
NI	–	–	7	15
I	–	3	7	15
Abs	1	–	7	15
I-Abs1	1	3	7	15
I-Abs3	3	3	7	15
I-Abs6	6	3	7	15

^1^ Bees were non-infected (NI) or infected with *N. ceranae* (I) and treated with *A. bisporus* extract (Abs). ^2^ Days after bee emergence.

**Table 2:** Primer used for qPCR analysis.

Primer	Sequence 5′–3′	Reference
Beta actin-F	TTGTATGCCAACACTGTCCTTT	[62]
Beta actin-R	TGGCGCGATGATCTTAATTT	
Abaecin-F	CAGCATTCGCATACGTACCA	[63]
Abaecin-R	GACCAGGAAACGTTGGAAAC	
Hymenopt-F	CTCTTCTGTGCCGTTGCATA	[63]
Hymenopt-R	GCGTCTCCTGTCATTCCATT	
Defensin-F	TGCGCTGCTAACTGTCTCAG	[63]
Defensin-R	AATGGCACTTAACCGAAACG	
ApidNT-F	TTTTGCCTTAGCAATTCTTGTTG	[62]
ApidNT-R	GTAGGTCGAGTAGGCGGATCT	
VgMC-F	AGTTCCGACCGACGACGA	[62]
VgMC-R	TTCCCTCCCACGGAGTCC	

## Data Availability

The data presented in this study are available on request from the corresponding author. The data are not publicly available due to the excessive data size.

## Supplementary Materials

The following are available online at https://www.mdpi.com/article/10.3390/insects12100915/s1, Figure S1: Daily food consumption per bee in the non-infected group (NI), infected group (I) and the groups infected with *N. ceranae* and treated with *A. bisporus* extract from the day 1 (I-Abs1), day 3 (I-Abs3) and day 6 (I-Abs6).
