# Supplementary material for: Effects of Agaricus bisporus Mushroom Extract on Honey Bees Infected with Nosema ceranae
Source: Insects. 2021 Oct 7;12(10):915. doi: 10.3390/insects12100915 (PMC8541333; doi:10.3390/insects12100915)
Supplement: Supplementary file 1 [file insects-12-00915-s001.zip › insects-1380777-supplementary.pdf]

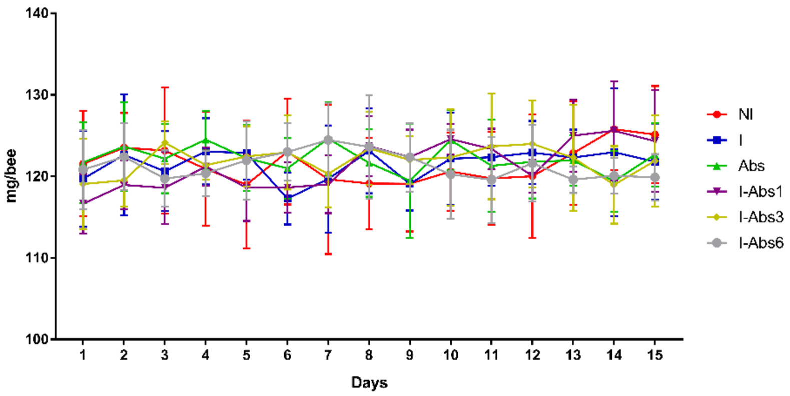

Figure S1. Daily food consumption per bee in the non-infected group (NI), infected group (I), and the groups infected with *N. ceranae* and treated with *A. bisporus* extract from day 1 (I-Abs1), day 3 (I-Abs3), and day 6 (I-Abs6).
